# A‐kinase‐interacting protein 1 facilitates growth and metastasis of gastric cancer cells via Slug‐induced epithelial‐mesenchymal transition

**DOI:** 10.1111/jcmm.14339

**Published:** 2019-04-24

**Authors:** Dehu Chen, Gan Cao, Qinghong Liu

**Affiliations:** ^1^ Department of General Surgery Taizhou People's Hospital The Fifth Affiliated Hospital of Nantong University Taizhou China

**Keywords:** AKIP1, epithelial‐mensenchymal transition, gastric cancer

## Abstract

A‐kinase‐interacting protein 1 (AKIP1) has previously been reported to act as a potential oncogenic protein in various cancers. The clinical significance and biological role of AKIP1 in gastric cancer (GC) is, however, still elusive. Herein, this study aimed to investigate the functional and molecular mechanism by which AKIP1 influences GC. AKIP1 mRNA and protein expressions in GC tissues were examined by quantitative real‐time PCR (qRT‐PCR), Western blot and immunohistochemistry. Other methods including stably transfected against AKIP1 into gastric cancer cells, wound healing, transwell assays, CCK‐8, colony formation, qRT‐PCR and Western blot in vitro and tumorigenesis in vivo were also performed. The up‐regulated expression of AKIP1 in GC specimens significantly correlated with clinical metastasis and poor prognosis in patients with GC. AKIP1 knockdown markedly suppressed GC cells proliferation, invasion and metastasis both in vitro and in vivo. In contrast, AKIP1 overexpression resulted in the opposite effects. Moreover, mechanistic analyses indicated that Slug‐induced epithelial‐mesenchymal transition (EMT) might be responsible for AKIP1‐influenced GC cells behaviour. Our findings demonstrated that high AKIP1 expression significantly correlated with clinical metastasis and unfavourable prognosis in patients with GC. Additionally, AKIP1 promoted GC cells proliferation, migration and invasion by activating Slug‐induced EMT.

## 1 INTRODUCTION

Gastric cancer (GC) is one of the most lethal malignancies worldwide.^1^ Despite great improvements in surgical treatment and adjuvant therapy in recent decades, long‐term survival remains unsatisfactory on account of tumour recurrence and metastasis.^2^, ^3^ Hence, the investigation of the molecular mechanisms underlying GC progression and metastasis contributes to identifying novel therapeutic targets, which is critical to improving GC treatment outcomes.

Recently, extensive evidence has demonstrated that epithelial‐mesenchymal transition (EMT) functions importantly in the invasion and metastasis of various epithelial tumours.^4^, ^5^, ^6^ The differentiation process that epithelial cells can down‐regulate epithelial properties and acquire mesenchymal features is commonly known as EMT. During this event, epithelial cells depolarize, lose their cell‐cell junctions and gain migratory and invasive potential, with the reduced expression of epithelial marker E‐cadherin and the increased expression of mesenchymal marker N‐cadherin.^4^ Regulation of EMT correlates with aberrant expression of usually repressed transcriptional factors such as Snail, Slug, ZEB1, SIP1 and Twist. Besides, several signalling pathways, including TGFβ, Notch, Wnt and PI3K/AKT signalling cascade, activated in the EMT evolution are essential regulators of EMT.^4^ Since accumulating evidences have unveiled the potential clinical value of targeting EMT in cancer treatment,^7^ we focus here on the molecular mechanisms by which certain genes control EMT in GC progression.

A‐kinase‐interacting protein 1 (AKIP1), a small 23‐kDa protein, is also named breast cancer‐associated gene 3, which encodes an alternatively spliced proline‐rich protein.^8^ Recent studies have indicated that AKIP1, identified as a differentially expressed gene, was elevated in many human malignancies, and might be involved in tumorigenesis and invasiveness, such as non‐small‐cell lung cancer and breast cancer, suggesting that AKIP1 could be exploited as a target for cancer treatment.^9^, ^10^ However, to the best of our knowledge, the clinicopathological and biological roles of AKIP1 in GC has not yet been elucidated.

In this study, we therefore investigated the clinical significance of AKIP1 in GC tissues, as well as the function and the underlying mechanism of AKIP1 in GC cells growth, migration and invasion.

## 2 MATERIALS AND METHODS

### 2.1 Patients, specimens and cell lines

A total of 96 patients with GC who underwent radical resection at the Fifth Affiliated Hospital of Nantong University were enrolled in this study. None of these participants was exposed to preoperative chemotherapy or radiotherapy. Among them, fresh tissues of 50 cases were performed for the detection of AKIP1 mRNA and protein expressions. In addition, another cohort of 96 cases was used for immunohistochemistry (IHC) evaluation. Written informed consent was obtained from all patients. The study was approved by the Ethics Committee of the Fifth Affiliated Hospital of Nantong University, and was carried out according to the World Medical Association Declaration of Helsinki.

The human GC cell lines (SGC7901, MKN45, AGS, MGC803 and MKN28) and normal gastric epithelial GES‐1 cells were purchased from the Type Culture Collection of the Chinese Academy of Sciences (Shanghai, China). All cells were maintained in RPMI‐1640 medium supplemented with 10% FBS (Gibco, USA) at 37°C with 5% CO_2_.

### 2.2 Lentivirus infection

Stable cell lines expressing shAKIP1 or AKIP1 were generated by transfection of pSuper‐shAKIP1 or pMSCV‐AKIP1.^11^ Transfections were carried out using Lipofectamine 2000 (Invitrogen, USA) in accordance with the manufacturer's protocols. The transfection efficiency was determined by Western blot analysis.

### 2.3 RNA isolation and quantitative real‐time polymerase chain reaction (qRT‐PCR)

Total RNA was extracted from tissues or cells using Trizol reagent (Invitrogen, USA). The complementary DNA (cDNA) was synthesized using the reverse transcription kit (TaKaRa, Japan) following the manufacturer's description. Quantitative real‐time polymerase chain reaction (qRT‐PCR) analysis was conducted using SYBR Green assay kit (Takara, China) on a 7500 RT‐PCR system (Applied Biosystems, USA). The 2^−ΔΔCt^ method was performed for relative quantification. The primers are listed in Supplementary Material.

### 2.4 Western blot

The approach for Western blot was conducted as described previously.^12^ Primary antibody against AKIP1 was bought from Abcam (UK). Primary antibodies against E‐cadherin, Snail, Slug, ZEB1, SIP1, Twist and N‐cadherin were obtained from CST (USA). GAPDH antibody, employed as the loading control, was purchased from Bioworld Technology (USA). Proteins were visualized with an enhanced chemiluminescence detection reagent (Thermo scientific, USA).

### 2.5 Immunohistochemistry

IHC was performed using standard procedures as described previously.^13^ Antibodies used for IHC analysis were AKIP1 (Abcam, UK), Slug (CST, USA) and E‐cadherin (CST, USA). Quantification of the IHC scores was performed by two independent pathologists (double‐blinded). The final staining scores were graded as reported previously.^13^

### 2.6 Wound healing assay

Cells were added to a six‐well plate, and then cultured to a confluent monolayer. A linear scratch was gently made using a sterile 10‐µl plastic tip, followed by an observation of the distance migrated by the cells at the indicated time (0 hour and 48 hour) to monitor the wound healing process. The percentage recovering of the wound = (0 hour width − 48 hour width)/0 hour width × 100%.

### 2.7 Cells migration and invasion assays

For migration assay, cells were plated into a 24‐well 8‐µm pore size transwell chamber (Corning, USA). For invasion assay, transwell chamber was coated with Matrigel (BD Biosciences, USA). For cells migration and invasion assays, the detailed conditions were described previously.^14^

### 2.8 Cell proliferation assay

Cells were seeded in a 96‐well plate. Ten microlitres of cell counting kit‐8 (CCK‐8, Dojindo, Japan) reagent was added to each well at the appropriate times (0, 24, 48, 72 and 96h after culture), followed by a detection of the absorbance value at 450 nm to analyse cell viability.

### 2.9 Colony formation assay

Cells were added in a six‐well plate. Following incubation at 37°C in a 5% CO_2_ humidified incubator for 2 weeks, cells were stained with crystal violet solution, and imaged under a microscope. Clone formation was defined as those containing ≥50 cells.

### 2.10 Animal experiments

BALB/c nude mice (six‐week‐old, male) were performed to assess GC cells tumorigenicity and metastasis in vivo. To characterize the effect of AKIP1 on tumour formation, cells were subcutaneously implanted into nude mice followed by measurement of tumour volume at 5‐day intervals using the formula: volume = (short diameter)^2^ × (long diameter)/2.^15^ At 30 days after inoculation, the tumours were weighed and sectioned for Western blot analysis. To figure out the role of AKIP1 in tumour metastasis, cells were injected intravenously into the tail vein of nude mice. The mice were killed at 30 days after injection, when the lungs were harvested for the count of metastasis nodules, and then processed for IHC and Western blot analysis. All animal work was carried out following the guidelines of the Ethics Committee of the Fifth Affiliated Hospital of Nantong University.

### 2.11 Statistical analysis

A chi‐square test was performed to analyse the association of AKIP1 expression with clinicopathological features. Kaplan‐Meier plot and Log‐rank test were used for survival analysis. All the data were presented as the means ± SD, and were analysed by Student's *t* test. All statistical analyses were performed using SPSS 21.0 software (SPSS Inc, USA). A value of *P* < 0.05 was considered statistically significant.

## 3 RESULTS

### 3.1 Clinical significance of AKIP1 in patients with GC

**Figure 1:**
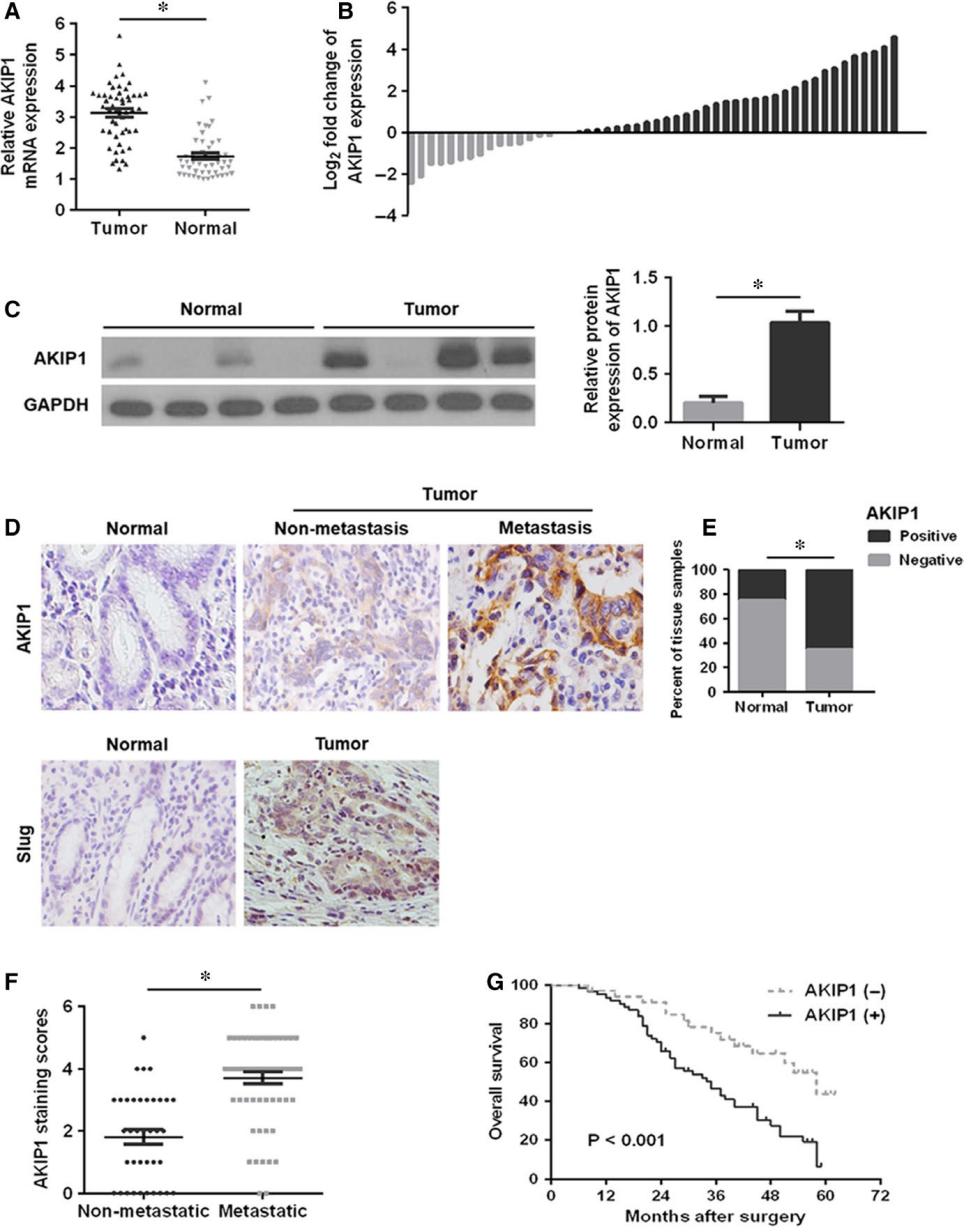
Relative A‐kinase‐interacting protein 1 (AKIP1) expression in gastric cancer (GC) tissues and its clinical significance. (A, B) Quantitative real‐time PCR analysis of AKIP1 mRNA expression in GC tumour tissues and corresponding normal tissues. (C) Western blot analysis of AKIP1 protein expression in GC tumour tissues and matched normal tissues. (D) Representative immunohistochemistry images of AKIP1 and Slug in GC tissues and adjacent normal tissues. (E) Quantitative assessment of AKIP1 expression in tumour tissues and matched normal tissues in accordance with staining scores. (F) Scatterplot of the staining scores of AKIP1 expression in patients without or with metastasis. (G) Kaplan‐Meier survival curve for GC patients with AKIP1 expression. *P < 0.05

To determine the role of AKIP1 in GC tissues, the mRNA and protein expressions of AKIP1 in 50 pairs of GC tissues and matched normal tissues were detected by qRT‐PCR and Western blot. As shown in Figure 1A,B, compared with that in matched normal tissues, AKIP1 mRNA was significantly increased in GC tissues. The AKIP1 protein analysis indicated the similar result (Figure 1C). Besides, the IHC analysis of 96 cases further revealed that AKIP1 expression was markedly elevated in tumour tissues compared with that in corresponding normal tissues (Figure 1D,E), especially in metastatic tumour tissues (Figure 1D,F).

Next, the relationship between AKIP1 and clinicopathological variables demonstrated that AKIP1 was closely related with tumour size, T stage, pTNM stage and lymph node metastasis respectively (*P* < 0.05) (Table 1), suggesting that AKIP1 might function importantly in GC progression and metastasis. Additionally, Table 2 showed that the expression level of AKIP1 was positively related to that of Slug (Figure 1D; *P* < 0.001, contingency coefficient = 0.512) in GC tissues. Kaplan‐Meier survival analysis turned out that patients with AKIP1 positive had poorer prognosis than those with AKIP1 negative (*P* < 0.05) (Figure 1E).

**Table 1:** Correlation between AKIP1 expression and clinicopathological features in patients with gastric cancer

Parameters	*n*	AKIP1		*P*‐value
		Negative	Positive	
Age (y)				
≥60	68	24	44	0.969
<60	28	10	18	
Gender				
Male	66	26	40	0.227
Female	30	8	22	
Tumour size (cm)				
≥5	58	14	44	0.004
<5	38	20	18	
Lauren's classification				
Diffuse	28	7	21	0.171
Intestinal	68	27	41	
Lymphatic vessel invasion				
With	37	13	24	0.964
Without	59	21	38	
T stage				
T_1_ + T_2_	38	21	17	0.001
T_3_ + T_4_	58	13	45	
pTNM stage				
I + II	37	19	18	0.010
III + IV	59	15	44	
Lymph node metastasis				
With (N_1_ + N_2_+N_3_)	59	13	46	0.001
Without (N_0_)	37	21	16	

**Table 2:** Correlation analysis between AKIP1 expression and Slug expression in gastric cancer tissues by chi‐square test

	AKIP1				
	Positive	Negative	χ^2^	*P*‐value	C
Slug					
Positive	52	8	34.113	<0.001	0.512
Negative	10	26			

### 3.2 AKIP1 promotes GC cells growth, migration and invasion in vitro

**Figure 2:**
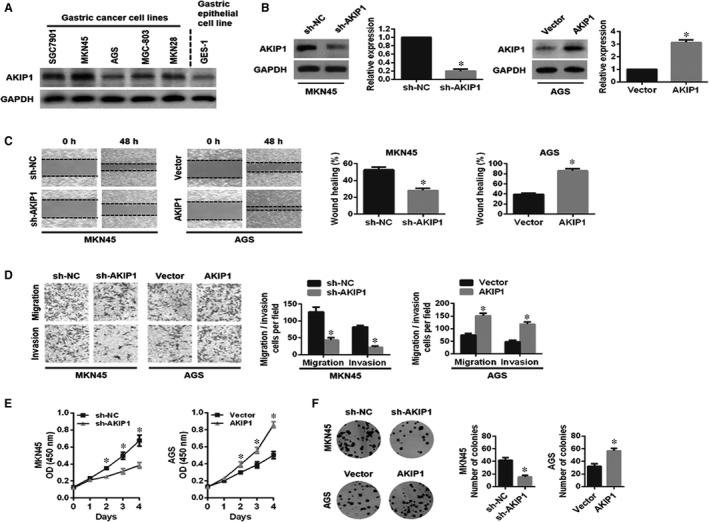
Influences of A‐kinase‐interacting protein 1 (AKIP1) knockdown or overexpression on gastric cancer (GC) cells migration, invasion and proliferation in vitro. (A) Western blot analysis of AKIP1 protein in GC cell lines and normal gastric epithelial GES‐1 cells. (B) The transfection effciency of AKIP1‐shRNA or AKIP1 overexpression was determined by Western blot analysis. (C, D) Influences of AKIP1‐altered expression on the migration and invasion capabilities of GC cells by wound healing assay and transwell assay. (E, F) Influence of AKIP1‐altered expression on the proliferation ability of GC cells by CCK‐8 assay and colony formation assay. *P < 0.05

To understand the function of AKIP1 in GC cells, we first selected the appropriate cells for a further study. As shown in Figure 2A, AKIP1 expression was the highest in MKN45 cells and was the lowest in AGS cells and GES‐1 cells, suggesting that AKIP1 expression was observably elevated in GC cells comparing with normal gastric epithelial GES‐1 cells. Subsequently, we found that AKIP1 expression was significantly decreased by shRNA‐AKIP1 in MKN45 cells, and notably increased by AKIP1 overexpression in AGS cells by Western blot analysis (Figure 2B).

Second, we examined the effect of AKIP1 expression on GC cells growth, migration and invasion in vitro. Both wound healing and transwell migration assays revealed that MKN45 cells migration was effectively inhibited by AKIP1 silencing, whereas AKIP1‐overexpressing AGS cells led to the greater migration capability (Figure 2C,D). Consistently, the transwell invasion assay demonstrated the similar effect on GC cells invasion (Figure 2D). Besides, we found that AKIP1 knockdown markedly suppressed MKN45 cells proliferation, while AKIP1 overexpression exerted the opposite result in AGS cells via CCK‐8 assay and colony formation assay (Figure 2E,F). Together, these data revealed that AKIP1 facilitated GC cells growth, migration and invasion in vitro.

### 3.3 Slug‐mediated EMT signalling activation is responsible for AKIP1‐induced GC cells growth, migration and invasion

**Figure 3:**
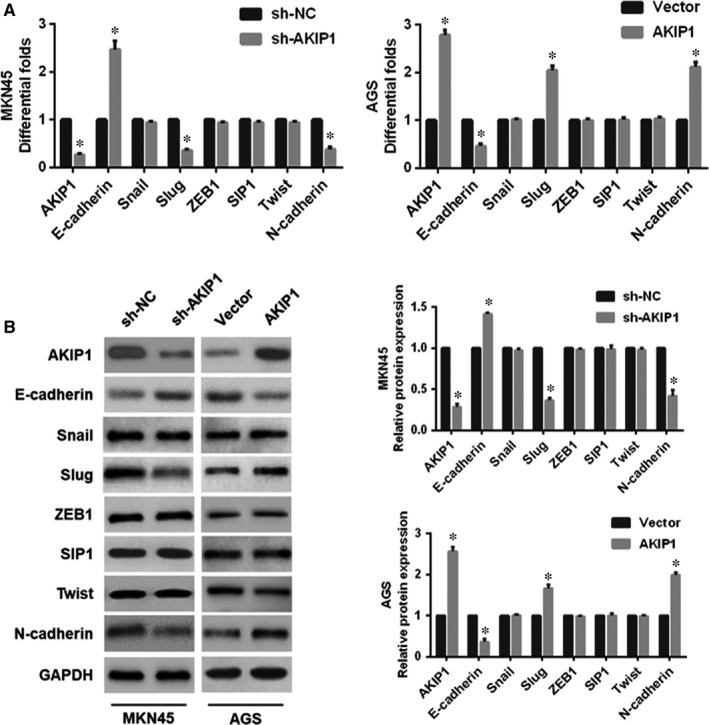
Effects of A‐kinase‐interacting protein 1 (AKIP1)‐altered expression on epithelial‐mesenchymal transition (EMT)‐related markers in gastric cancer cells. Following AKIP1 shRNA or overexpression treatment, quantitative real‐time PCR (A) and Western blot (B) were employed to evaluate the expressions of EMT‐related markers in AKIP1‐altered cells. *P < 0.05

Emerging evidence suggested that EMT functions importantly in cancer initiation, progression and metastasis.^16^ In this study, qRT‐PCR analysis revealed that AKIP1 silencing led to the up‐regulated expression of E‐cadherin and the down‐regulated expressions of N‐cadherin and Slug, but did not markedly influence other transcription factors (Snail, ZEB1, SIP1 and Twist). Conversely, AKIP1 overexpression exerted the opposite outcome (Figure 3A). Consistent with the qRT‐PCR finding, Western blot analysis further demonstrated the similar effect (Figure 3B). Based on these results, we hypothesized that AKIP1 might facilitate EMT by interacting with transcription factor Slug in GC cells.

**Figure 4:**
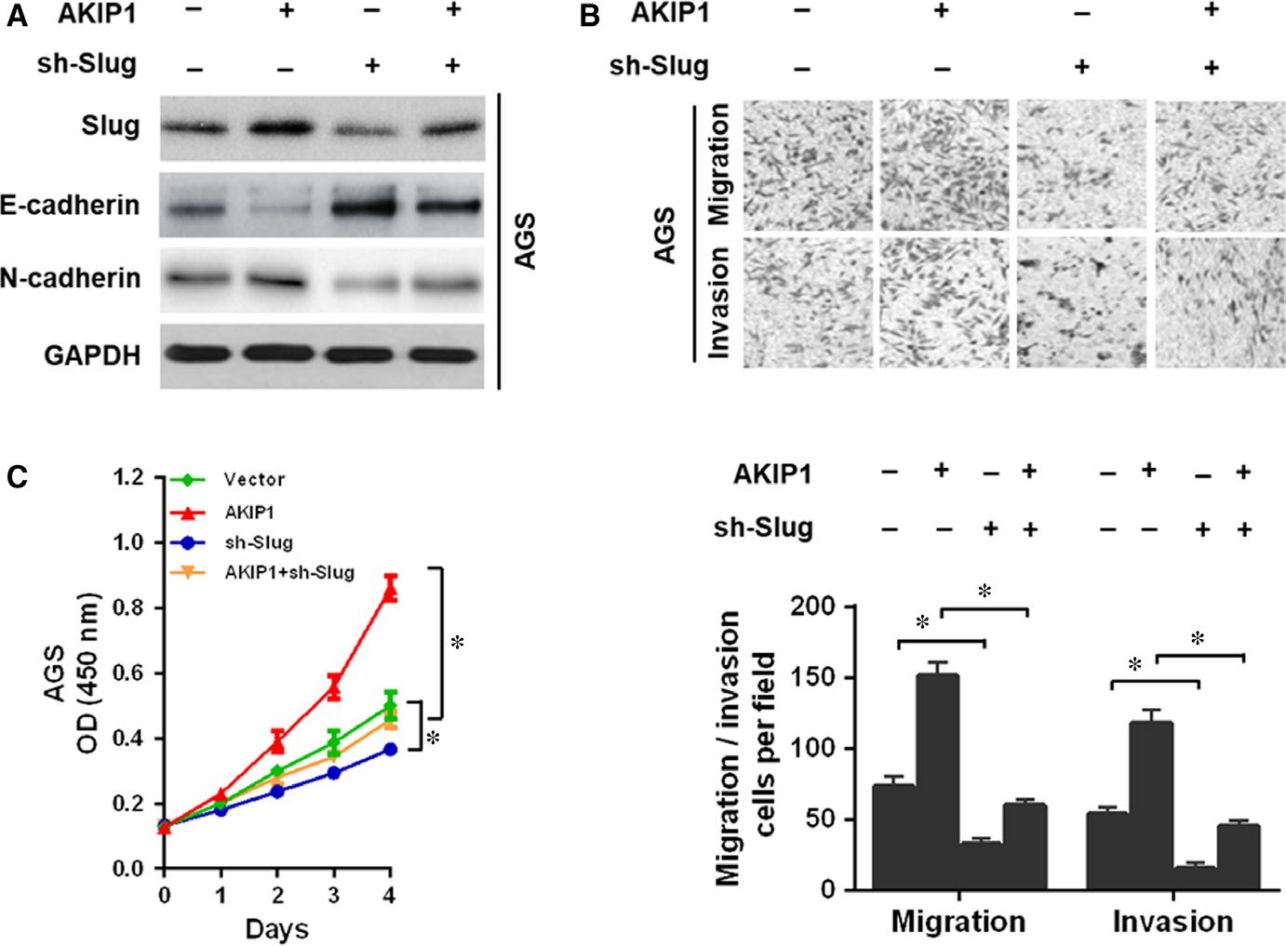
A‐kinase‐interacting protein 1 (AKIP1) facilitated gastric cancer cells migration, invasion, and growth via Slug‐mediated epithelial‐mesenchymal transition (EMT). (A) Downregulation of Slug converted AKIP1‐induced cells EMT by Western blot analysis. (B) Slug silencing reversed AKIP1‐facilitated cells migration and invasion by transwell assay. (C) Slug knockdown reversed AKIP1‐mediated cells growth by CCK‐8 assay. *P < 0.05

Next, to test the hypothesis, the Slug expression was suppressed in GC cells. According to the results, we found that Slug silencing in AGS cells resulted in an obvious reversal of AKIP1‐induced EMT (Figure 4A). Moreover, Slug knockdown in AGS cells notably suppressed AKIP1‐facilitated cell migration and invasion (Figure 4B), as well as cells growth (Figure 4C). In summary, these results implied that AKIP1 promoted GC cells growth, migration and invasion, at least in part, via the activation of Slug‐mediated EMT.

### 3.4 AKIP1 promotes tumour formation and metastatic potential in vivo

**Figure 5:**
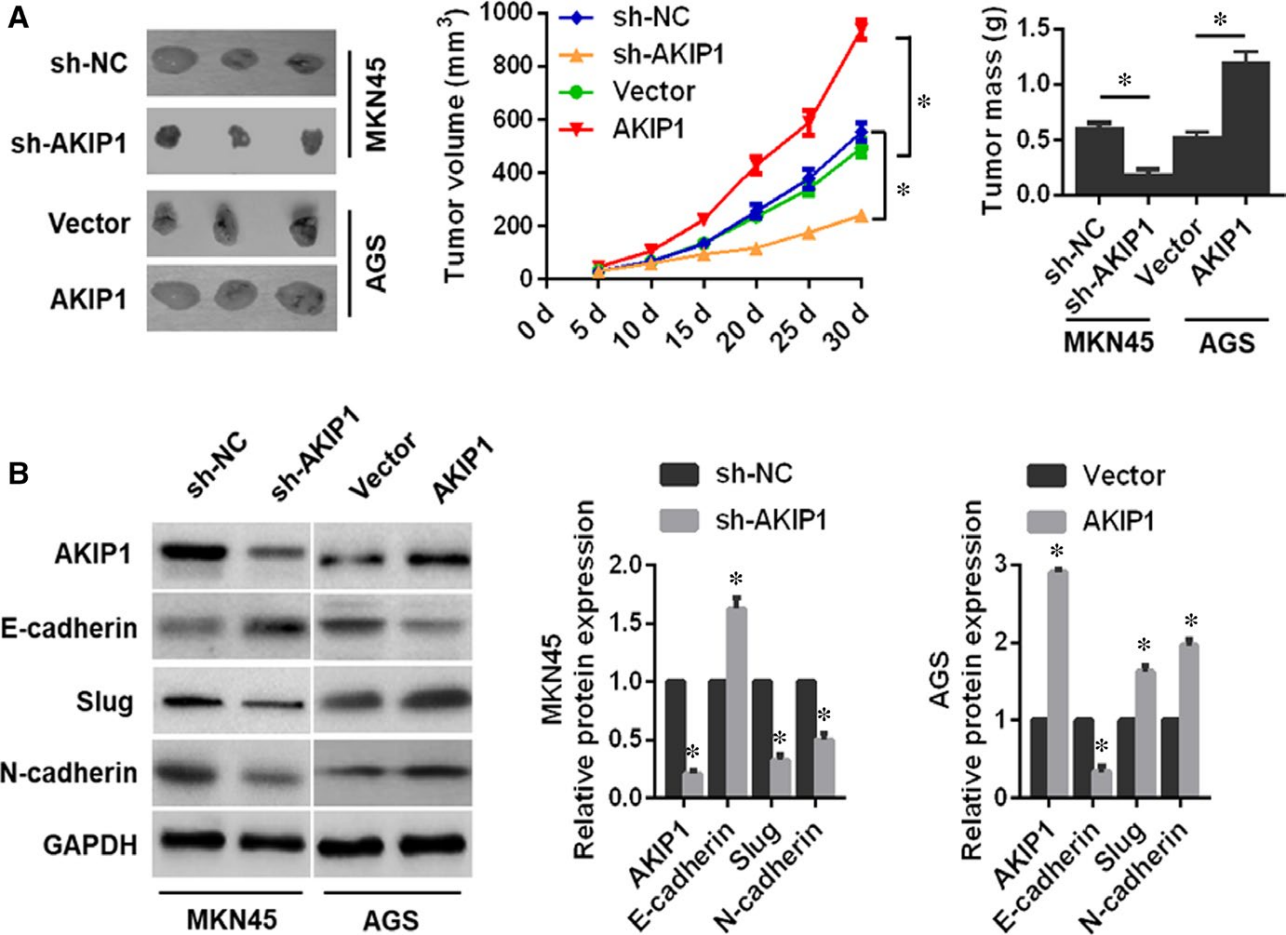
Influences of A‐kinase‐interacting protein 1 (AKIP1) knockdown or overexpression on gastric cancer (GC) cells tumour growth in vivo. (A) Representative images of xenografts in nude mice established via subcutaneous injection of GC cells. Tumour volume was calculated every 5 days. Xenograft tumours were harvested for weight. (B) Western blot analysis of expressions of AKIP1, E‐cadherin, Slug, and N‐cadherin in xenograft tumours. *n* = 6 in each group. *P < 0.05

To make a further verification of the role of AKIP1 in vivo, we established subcutaneous xenograft tumour and lung metastasis model in nude mice. After 30 days, MKN45 sh‐NC cell‐derived tumours at the subcutaneous implantation sites were larger and grew more rapidly than MKN45 sh‐AKIP1 cell‐derived tumours (Figure 5A). Consistent with the results in vitro, Western blot analysis revealed that AKIP1 depletion decreased the expressions of Slug and N‐cadherin, and increased the expression of E‐cadherin (Figure 5B). On the contrary, AKIP1 overexpression resulted in the opposite effects (Figure 5A,B).

**Figure 6:**
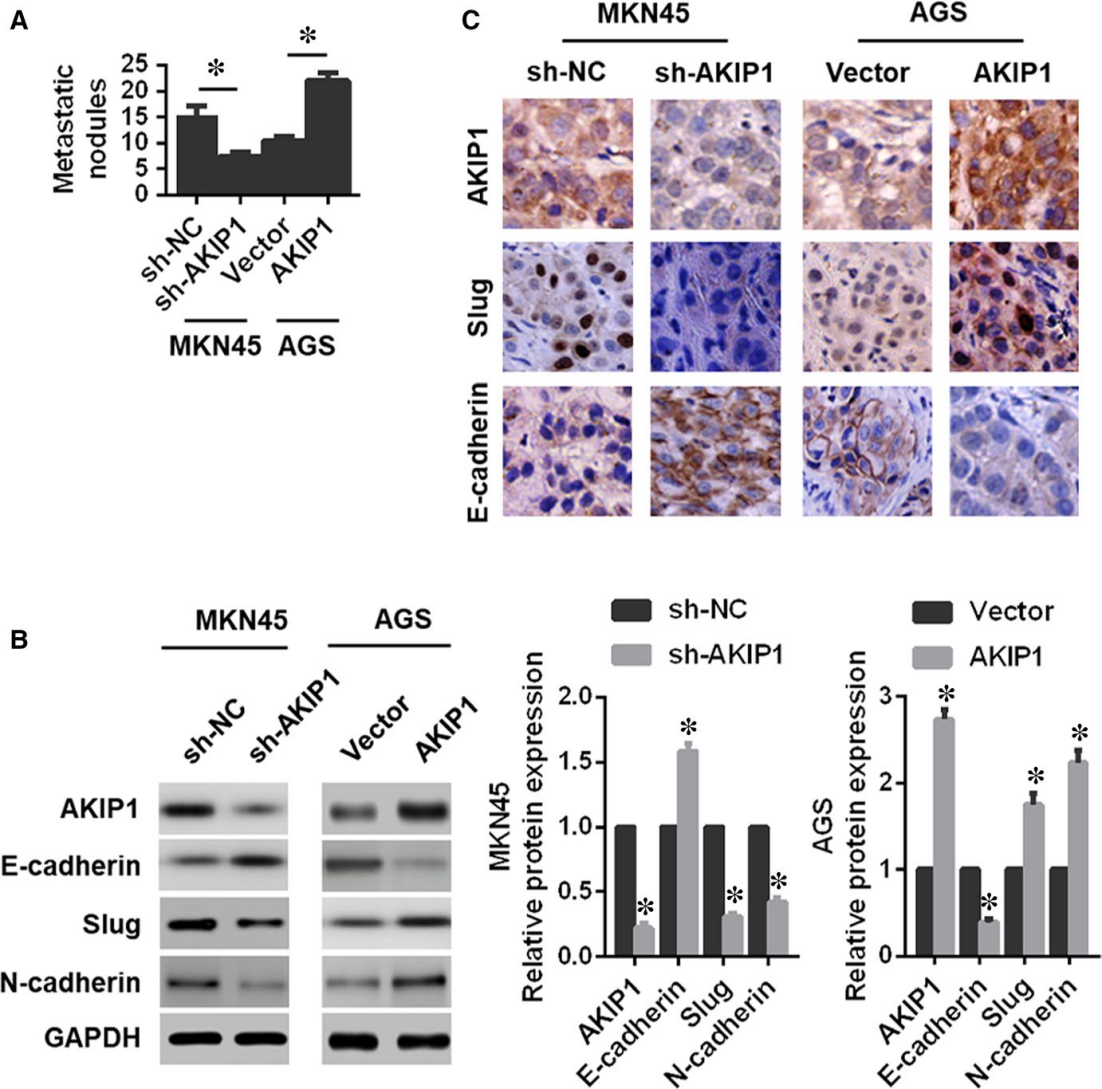
Effects of A‐kinase‐interacting protein 1 (AKIP1) knockdown or overexpression on the metastatic capability of gastric cancer cells in vivo. (A) The count of the lung metastatic nodules. (B) Western blot analysis of expressions of AKIP1, E‐cadherin, Slug, and N‐cadherin in the lung metastatic nodules. (C) Immunohistochemistry analysis of the expressions of AKIP1, Slug, and E‐cadherin in the lung metastatic nodules. *n* = 6 in each group. *P < 0.05

In addition, the lung metastasis model demonstrated that AKIP1 knockdown exhibited an attenuated lung metastasis (Figure 6A), and an obvious reversal of EMT (Figure 6B,C). As expected, AKIP1 overexpression led to a significantly opposite effect on cells metastasis in vivo (Figure 6A‐C). Consequently, these results suggested that AKIP1 could contribute to GC cells growth and metastasis in vivo.

## 4 DISCUSSION

Since recurrence and distant metastases remain the major reasons for death in cancer patients,^17^ great efforts are urgently needed to identify novel tumour markers for early detection and deeply understand the potential mechanisms involved in cancer cells progression for targeted therapies. Recent investigations indicated that AKIP1 dysregulation reflected physiological and pathological alterations in various types of cancer, and might be involved in tumour progression and metastasis, suggesting that AKIP1 might be a potent oncogenic gene.^9^, ^18^, ^19^ It is worth noting that AKIP1 expression was markedly elevated in oesophageal squamous cell carcinoma (ESCC) cell lines and clinical ESCC samples, strongly correlated with the clinical stage, T and N classifications, and clinical prognosis in ESCC patient.^11^ Another study in non‐small‐cell lung cancer (NSCLC) showed that AKIP1 could be a potential mediator of tumour metastasis, and might serve as a therapeutic target for NSCLC.^9^ Moreover, in breast cancer, Mo et al indicated that AKIP1 might significantly promote cellular motility and invasion via the activation of Akt/GSK‐3β/Snail pathway.^10^ Although AKIP1 has been considered as a critical regulator of proliferation and metastasis in several human malignancies, the consensus knowledge of its clinical significance, molecular functionality and novel mechanisms involved in tumour biological aggressiveness especially in GC has not yet been elucidated.

In this study, we demonstrated that AKIP1 was highly expressed in GC tissues compared with that in their non‐tumour counterparts. The clinical association analysis revealed overexpression of AKIP1 was positively correlated with tumour size, clinical metastasis, and a shorter OS time of GC patients, thus proving its crucial role in GC evolution and metastasis. Moreover, we investigated the biological roles of AKIP1 in GC cells. The results showed that AKIP1 depletion suppressed GC cells growth, migration and invasion both in vitro and in vivo, whereas AKIP1 overexpression resulted in the opposite effects. Mechanistic investigation revealed that AKIP1 promoted EMT in GC cells via activation of transcription factor Slug.

It is widely accepted that the initial step, acquisition of migration and invasion capability, from a relatively immobile type to a more invasive phenotype, is the rate‐limiting step in metastatic cascade.^16^, ^20^ As mentioned in the introduction, EMT is generally considered as a key event during the initial steps in cancer metastasis and progression. In consideration of the clinical significance of this evolution, the reversal of EMT becomes a promising therapeutic approach designed to notably improve disease outcome. Considering that the spread of cancer cells to distant organs, from a relatively immobile type to a more invasive phenotype, is generally considered as a key event during cancer progression and metastasis, novel effective strategies are urgently needed to restrain the metastatic dissemination of cancer cells. Remarkably, our findings manifested that AKIP1 knockdown reversed EMT process, whereas AKIP1 overexpression accelerated EMT evolution, suggesting AKIP1 as a crucial modulator of EMT in GC cells. Interestingly, transcription factor (such as Snail, Slug, ZEB1, SIP1 and Twist), as a key regulators of EMT, was found to be an intracellular element related to cellular proliferation, invasion and metastasis.^21^ Herein, we discovered the role of Slug in AKIP1‐mediated regulation of GC proliferation and metastasis. The results demonstrated that AKIP1 silencing decreased Slug expression, whereas AKIP1 overexpression promoted Slug expression. Also, knockdown of the endogenous Slug expression weakened not only AKIP1‐induced EMT, but also cells growth, invasion and metastasis. More importantly, Clinically, AKIP1 expression was positively correlated with Slug expression in GC tissues. Hence, we could reasonably assume that AKIP1 promoted GC cells growth, invasion and metastasis by activating Slug‐induced EMT.

However, it is worth further exploring the indirect or direct relationship between AKIP1 and Slug. Additionally, as reported, the NF‐κB signalling, Akt/GSK‐3β/Snail signalling or transcription factor ZEB1 was also demonstrated to function importantly in AKIP1‐mediated tumour progression and metastasis.^9^, ^10^, ^11^ Consequently, we will further assess whether other genes or certain signalling pathways are involved in AKIP1‐induced EMT in subsequent studies.

Taken together, these results revealed that high AKIP1 expression significantly correlated with clinical metastasis and unfavourable prognosis in patients with GC, and AKIP1 facilitated GC cells growth, invasion and metastasis by activating Slug‐induced EMT. Thus, repressing AKIP1 and EMT inducers such as Slug have potential clinical applications for GC treatment.

## CONFLICT OF INTEREST

The authors confirm that there are no conflicts of interest.
