# Supplementary material for: A‐kinase‐interacting protein 1 facilitates growth and metastasis of gastric cancer cells via Slug‐induced epithelial‐mesenchymal transition
Source: J Cell Mol Med. 2019 Apr 24;23(6):4434–42. doi: 10.1111/jcmm.14339 (PMC6533465; doi:10.1111/jcmm.14339)
Supplement: Supplementary file 1 [file JCMM-23-4434-s001.doc]

**Supplementary 1. Primers designed for qRT-PCR**

| **Genes** | **Sequence (5’-3’)** | |
| --- | --- | --- |
|  | **Forward** | **Reverse** |
| AKIP1 | CATGGACAACTGTTTGGCGG | CTGTTTCTCTAGGTGGGGCG |
| E-cadherin | CGAGAGCTACACGTTCACGG | GGGTGTCGAGGGAAAAATAGG |
| N-cadherin | TCAGGCGTCTGTAGAGGCTT | ATGCACATCCTTCGATAAGACTG |
| Snail | AAGGCCTTCTCTAGGCCCT | CGCAGGTTGGAGCGGTCAG |
| Slug | TTCGGACCCACACATTACCT | GCAGTGAGGGCAAGAAAAAG |
| ZEB1 | GATGATGAATGCGAGTCAGATGC | ACAGCAGTGTCTTGTTGTTGT |
| SIP1 | CAAGAGGCGCAAACAAGCC | GGTTGGCAATACCGTCATCC |
| Twist | CAGCTACGCCTTCTCGGTCT | CTGTCCATTTTCTCCTTCTCTGGA |
| GAPDH | AGGGGCCATCCACAGTCTTC | AGAAGGCTGGGGCTCATTTG |
